# Long-term multicenter comparison shows equivalent efficacy of monoclonal antibodies in severe asthma therapy

**DOI:** 10.1186/s12890-024-02964-4

**Published:** 2024-03-21

**Authors:** Moritz Z. Kayser, Hendrik Suhling, Jan Fuge, Christopher A. Hinze, Nora Drick, Nikolaus Kneidinger, Jürgen Behr, Christian Taube, Tobias Welte, Ina Haasler, Katrin Milger

**Affiliations:** 1 Department of Respiratory Medicine and Infectious Disease, Hannover Medical School, Carl-Neuberg-Str. 1, 30625 Hannover, Lower Saxony Germany; 2 Biomedical Research in Endstage and Obstructive Lung Disease Hannover (BREATH), Member of the German Center for Lung Research (DZL), Hannover, Germany; 3 Department of Medicine V, University Hospital, LMU, Munich, Germany; 4 Comprehensive Pneumology Center Munich (CPC-M), Member of the German Center for Lung Research (DZL), Munich, Germany; 5 Department of Pulmonary Medicine, University Hospital Essen-Ruhrlandklinik, Essen, Germany

**Keywords:** Severe asthma, Long-term treatment response, Mepolizumab, Benralizumab, Dupilumab, Biologic Asthma Response Score

## Abstract

**Background:**

Monoclonal antibodies (biologics) drastically changed severe asthma therapy. Mepolizumab (anti-interleukin (IL) 5), benralizumab (anti-IL5 receptor alpha), and dupilumab (anti-IL4/13) are the most used biologics in this context. While all biologics are efficient individually, the choice of biologic is complicated by insufficient data on their comparative long-term treatment efficacy. Here, we compare the real-life efficacy of these biologics in asthma therapy over 12 months.

**Methods:**

280 severe asthma patients treated with mepolizumab (129/280, 46%), benralizumab (83/280, 30%) or dupilumab (68/280, 24%) for one year were analyzed retrospectively. Data were collected at baseline and after 6 and 12 months of therapy. Endpoints were changes pulmonary function (PF), exacerbation rate, oral corticosteroid (OCS) use and dose, asthma control test (ACT) score and fractional exhaled nitric oxide (FeNO) levels as well as responder status measured by the recently published “Biologic Asthma Response Score” (BARS).

**Results:**

All biologics led to significant improvements in PF, ACT and OCS dose. Only Mepolizumab and Benralizumab significantly decreased the exacerbation rate, while only Mepolizumab and Dupilumab significantly decreased FeNO. Responder rates measured by BARS were high across all groups: roughly half of all patients achieved full response and most of the remainder achieved at least partial responder status. Overall, outcomes were similar between groups after both 6 and 12 months.

**Conclusions:**

All biologics showed great efficacy in individual parameters and high responder rates measured by BARS without a clinically relevant advantage for any antibody. Response was usually achieved after 6 months and retained at 12 months, emphasizing the utility of early response assessment.

**Supplementary Information:**

The online version contains supplementary material available at 10.1186/s12890-024-02964-4.

## Introduction

Severe asthma accounts for 5–10% of all asthma cases and is typically driven by type-2 inflammatory mediators [1]. Type-2 inflammation is characterized by eosinophilia in both peripheral blood and sputum as an expression of the eosinophils as one of the main effector cells in this form of airway inflammation [2–5]. A key causative factor in this inflammatory process are elevated levels of pro-inflammatory interleukins (IL)-4, -5 and -13 [5, 6]. Exacerbations are frequent and often severe in these patients, causing a high burden of direct and indirect morbidity and health-care associated costs [7–9]. Beyond recurrent exacerbations, the underlying type-2 inflammation may lead to airway remodeling and permanent loss of lung function [10].

Treatment of type-2 asthma has been revolutionized by the development of monoclonal antibodies (hereafter called biologics) targeting the underlying inflammatory pathways. These include the IL5-targeting biologics mepolizumab and reslizumab, the IL5 receptor alpha (IL5Rα)-targeting benralizumab, the IL4 and IL 13-targeting dupilumab and most recently, tezepelumab, targeting thymic stromal lymphopoietin (TSLP) [11, 12]. While all biologics individually demonstrated both good efficacy and safety profiles, there is a notable absence of head-to-head comparisons of the various biologics. Indirect comparisons of licensing trial data as well as retrospective comparisons of biologic efficacy by our groups and others have failed to show an advantage for any one biologic [13–21]. Notably, specific switches from one biologic to another have shown additional benefits in therapy non-responders, yet the ideal first-choice biologic in severe asthma remains unclear. Additionally, comparisons of biologics were up until now hampered by the absence of universal criteria for biologic therapy response, as endpoints differed both in licensing trials and retrospective analyses. Recently, a German group of experts published the Biologics Asthma Response Score (BARS), allowing an objective evaluation of therapy response based on exacerbation rate, asthma control (ACT) score and daily oral corticosteroid (OCS) dose [22, 23]. Using this novel scoring system and influenced by the steadily growing portfolio of biologics for the treatment of severe asthma, we present data on the efficacy of mepolizumab, benralizumab and dupilumab in the long-term treatment of severe asthma in this multi-center, retrospective analysis.

## Materials and methods

### Aim, design and setting

This multicenter, retrospective analysis compared treatment outcomes in patients with severe asthma treated with either the IL5 antibody mepolizumab, IL5Rα antibody benralizumab or the IL4/13 antibody dupilumab using BARS as well as individual factors like asthma control, pulmonary function (PF), exacerbation rate and OCS use and dosage over a 12-month period. All patients were treated between February 2016 and September 2022 at one of our university outpatient clinics in Germany (Essen, Munich and Hannover). This study was performed in accordance with the ethical guidelines of the 1975 Declaration of Helsinki and was approved by the ethics committees of all three participating university centers (Hannover 10008_BO_K_2021, Essen 21–10,369-BO, Munich 21–0436). Data were pseudonymized and the study relied exclusively on information collected as part of routine care.

### Inclusion criteria and treatment

All included patients had been diagnosed with severe asthma with inadequate asthma control despite a combination of medium to high-dose inhaled glucocorticoids and long-acting β2-agonist plus an optional second or third controller and / or additional oral corticosteroids (OCS). Additionally, prescription criteria for biologics in Germany require a blood eosinophil count of > / = 150 per µl at initiation or > / = 300 per µl within 12 months prior for mepolizumab and benralizumab or either a blood eosinophil count > / = 150 eosinophils/µl, an Exhaled nitric oxide (FeNO) of > 25 parts per billion or a need for permanent OCS for dupilumab (Supplementary Table 1). Consequently, a subcutaneous add-on therapy was initiated using either mepolizumab 100 mg once every 4 weeks, benralizumab 30 mg once every 4 weeks for the first 3 doses and once every 8 weeks for subsequent doses or dupilumab 600 mg for the initial dose followed by 200 mg or 300 mg every 2 weeks as indicated for subsequent doses [24]. The decision to start a biologic and the choice of biologic was made by the treating physician solely on clinical grounds. Patients were included in the present analysis if they had received biologic therapy for 12 months without interruption at one of the study sites. Patients who had previously received another biologic therapy were ineligible for study inclusion unless there was a wash-out phase of 6 months or longer between therapies. Non-antibody asthma medication and OCS therapy were adjusted over the course of biologic therapy by the treating physicians depending on the individual patients’ clinical situation.

### Data collection

Data was collected at three time points: at baseline (bl), up to 3 months prior to biologic therapy initiation, control timepoint 1 (timepoint 1, T1) 6 months ± 90 days after initiation and control timepoint 2 (timepoint 2, T2) 12 months ± 90 days after initiation. The latest suitable visit within the ± 90-day interval for a timepoint was used if more than one visit occurred within the interval.

FeNO, differential blood cell counts as well as forced expiratory capacity in 1 s (FEV1), functional vital capacity (FVC), residual volume (RV) and total lung capacity (TLC) were collected as part of routine follow-up care. PF was performed using ERS/ATS-standardized spirometry or body plethysmography [25]. Asthma control was assessed through the asthma control test (ACT) [26]. Changes in medication, exacerbation rates and ACT scores were re-assessed at each timepoint, with exacerbation rates being annualized to the 12 months prior. We defined exacerbations as an acute aggravation of asthma symptoms requiring de novo OCS or an increase in the OCS dose for at least 3 days [27]. Smoking status and atopic and eosinophilic phenotype-related comorbidities were assessed at baseline. BARS was calculated retrospectively for each patients at 6 and 12 months of therapy. To summarize, BARS evaluates therapy response based on changes in ACT, OCS dose and exacerbation rate, with 2, 1 or 0 points attainable in each category. A higher score corresponds to a better therapy response, with the overall response being assessed using the average of all three categories, allowing for categorization of therapy response into one of three categories (“good response”, “intermediate responders” and “insufficient response”) [22].

### Statistical analysis

Statistical analyses and figure preparation were performed using IBM SPSS v28™ (IBM SPSS Statistics, Armonk, NY) and R environment for statistical computing version 4.1.2 (R Foundation for Statistical Computing, Vienna, Austria). Categorical variables are stated as numbers (n) and percentages (%). Depending on distribution, continuous variables are shown as mean ± standard deviation (SD) or median with interquartile ranges (IQR) unless indicated otherwise. For group comparisons, Chi-squared test, two-sided ANOVA, Wilcoxon rank-sign, Mann–Whitney-U-test or t-test were used, as appropriate. All reported p-values are two-sided. *P*-values < 0.05 were considered statistically significant.

## Results

Data from 280 patients who had been treated with either mepolizumab (129/280, 46%), benralizumab (83/280, 30%) or dupilumab (68/280, 24%) for at least one year were analyzed. Baseline characteristics for all treatment groups were similar regarding the distribution of sex, age, baseline PF, ACT scores and OCS dose. However, the dupilumab cohort featured more patients suffering from atopic dermatitis, allergic rhinitis, and Aspirin (ASS) intolerance and contained more patients suffering from a mixed asthma phenotype, compared to a higher prevalence of purely eosinophilic asthma phenotypes among the mepolizumab and benralizumab cohorts. In addition, the dupilumab cohort experienced on average fewer yearly asthma exacerbations prior to initiation of therapy (Table 1).

**Table 1:** Patients baseline characteristics

	**Mepolizumab, *n* = 129**	**Benralizumab, *n* = 83**	**Dupilumab, *n* = 68**	**Inter-group comparison** ***p*-value**
Female sex, n (%)	76 (59%)	49 (59%)	33 (49%)	0.320^b^
Age, median (IQR)	56 (48 – 62)	60 (50 – 67)	56 (51 – 64)	0.197^a^
**Comorbidities**				
CRS, n (%)	13 (10%)	13 (16%)	4 (6%)	0.143^b^
CRSwNP, n (%)	34 (26%)	16 (19%)	14 (21%)	0.405^b^
Atopic Dermatitis, n (%)	6 (5%)	4 (5%)	21 (31%)	** < 0.001**^b^
Allergic Rhinitis, n (%)	4 (3%)	2 (2%)	10 (15%)	**0.001**^b^
ASS intolerance, n (%)	17 (13%)	7 (8%)	17 (25%)	**0.016**^b^
Asthma phenotype				
Allergic, n (%)	6 (5%)	4 (5%)	5 (7%)	**0.020**^b^
Eosinophilic, n (%)	98 (76%)	65 (78%)	38 (56%)	
Mixed, n (%)	25 (19%)	14 (17%)	25 (37%)	
**Smoking status**				
never smoked, n (%)	63 (49%)	42 (51%)	36 (53%)	0.812^b^
ex-smoker, n (%)	64 (50%)	41 (49%)	32 (46%)	
Pack years, median (IQR)	0 (0 – 20)	0 (0 – 10)	0 (0 – 6)	0.601^a^
**Baseline parameters**				
ACT score, median (IQR)	13 (10–19)	14 (10–20)	16 (11–19)	0.312^a^
Exacerbations per year, median (IQR)	1 (0 – 2)	1 (0 – 3)	0 (0 – 1)	**0.020**^a^
OCS-therapy, n (%)	72 (56%)	48 (58%)	29 (43%)	0.095^b^
OCS dose ^c^, median (IQR)	5 (0 – 10)	2.5 (0 – 7.5)	0 (0 – 5)	0.347^a^
FEV1% of predicted, median (IQR)	60 (44 – 79)	63 (47 – 80)	64 (48 – 82)	0.910^a^
FVC % of predicted, median (IQR)	82 (70 – 94)	79 (64 – 91)	81 (69 – 95)	0.496^a^
RV % of predicted, median (IQR)	140 (116 – 173)	136 (113 – 159)	134 (112 – 161)	0.290^a^
TLC % of predicted, median (IQR)	105 (95 – 116)	103 (92 – 109)	101 (93 – 112)	0.128^a^
FeNO ppb, median (IQR)	36 (19 – 71.3)	42.1 (24.1 – 65.2)	36 (21.1 – 56.4)	0.332^a^
Blood eosinophils per µl (IQR)	430 (70 – 740)	400 (63 – 678)	220 (70 – 475)	0.301^a^

*Abbreviations*: *ACT *Asthma control test, *ASS *Aspirin, *FEV1 *Forced expiratory capacity in 1 s, *CRS *Chronic rhinosinusitis, *CRSwNP *Chronic rhinosinusitis with nasal polyps, *FVC *Forced vital capacity, *IQR *Interquartile range, *NA N*ot available, *OCS *Oral corticosteroids

^a^Intergroup p-value calculated using two-way ANOVA with Bonferroni-correction

^b^Chi^2^-Test

^c^In mg prednisolone equivalent, *FeNO *Forced exhaled nitric oxygen, *ppb *Parts per billion, *IgE *Immunoglobulin

**Fig. 1:**
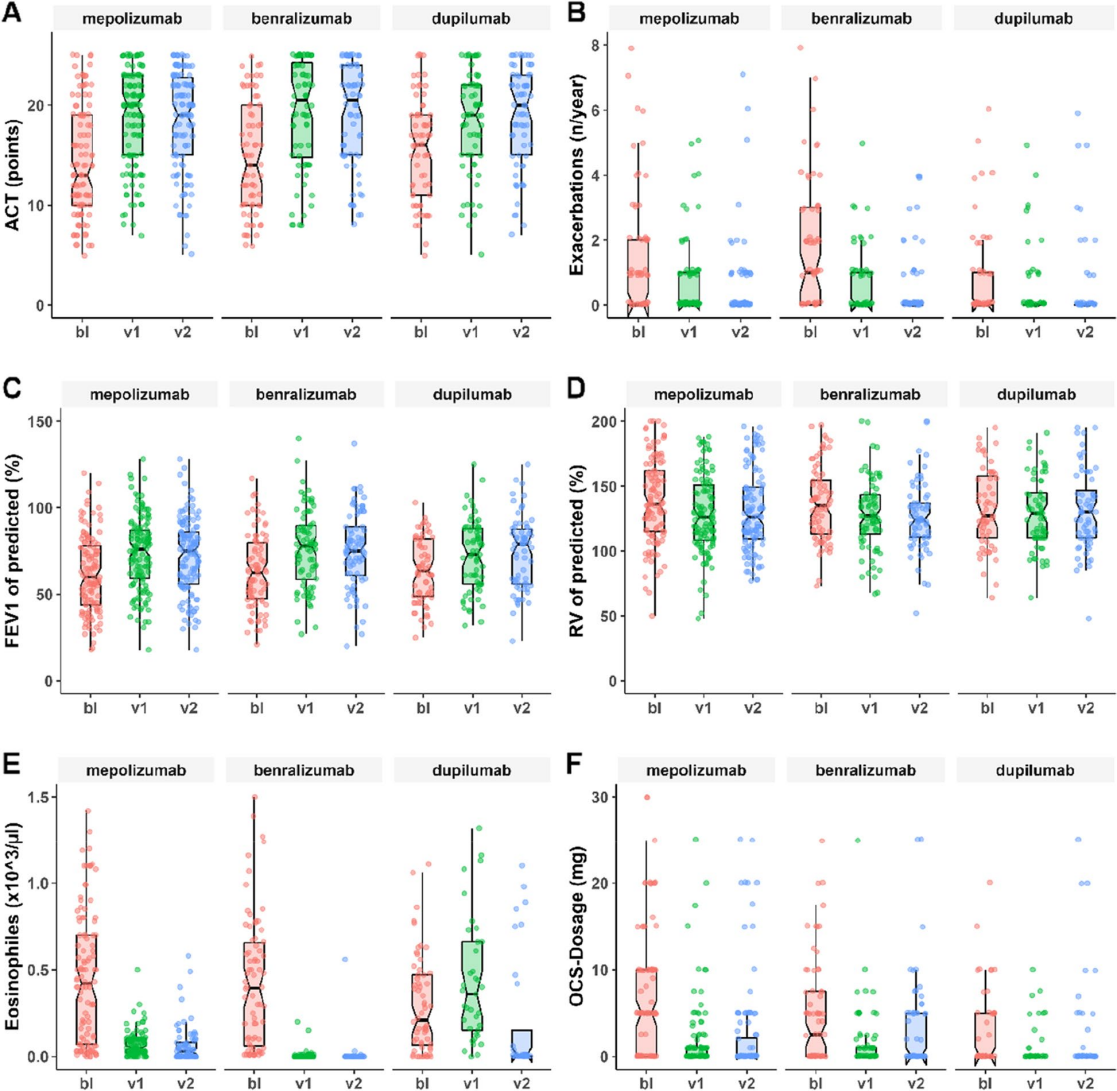
**A**-**F** Time course of ACT (**A**), Annualized exacerbation rate (**B**), FEV1% (**C**), RV% (**D**), Eosinophils (**E**) and oral corticosteroid dose (**F**). Notes: Graphs represent measurements per parameter and treatment group at baseline, T1 and T2 from left to right. Dark line indicates median, box indicates interquartile range, whiskers indicate minimum and maximum values. Abbreviations: BL – baseline, T1 – timepoint 1 (6 months), T2 – timepoint 2 (12 months), FEV1 (%) – percentage of forced expiratory capacity in 1 s compared to calculated normal, RV – percentage of residual volume compared to calculated normal, ACT – asthma control test, OCS – oral corticosteroids

Table 2 summarizes the changes in PF, ACT scores, exacerbation rates, OCS dose and laboratory values within groups between time points as well as the inter-group comparison of the changes (delta) in parameters from BL to T1 and T2. Asthma control assessed by the ACT improved significantly across all groups within the first 6 months of treatment and stayed well-controlled after 12 months (Table 2 and Fig. 1A). Nonetheless, there was a significantly larger improvement of ACT in patients receiving mepolizumab or benralizumab. The annualized exacerbation rate decreased significantly in patients receiving mepolizumab and benralizumab, but only trended towards a decrease (from a comparatively lower starting point) in patients receiving dupilumab (Fig. 1B). Consequently, we observed a significantly stronger reduction in exacerbations among the former two treatment groups than among the latter.

**Table 2:** Treatment outcomes over time

Median (IQR)	Mepolizumab, *n* = 129					Benralizumab *n* = 83					Dupilumab *n* = 68					Inter-group comparisons		
	**BL**	**T 1**	**p BL to T1**^a^	**T2**	**p BL to T2**^a^	**Baseline**	**T1**	**p BL to T1**^a^	**T2**	**p BL to T2**^a^	**Baseline**	**T1**	**p BL to T1**^a^	**T2**	**p BL to T2**^a^	**p delta BL to T1**^b^	**p delta T1 to T2**^b^	**p delta BL to T2**^b^
FEV1% of predicted	60 (44 – 79)	76 (59 – 88)	** < 0.001**	75 (56 – 87)	** < 0.001**	63 (47 – 80)	78 (58 – 90)	** < 0.001**	75 (60 – 89)	** < 0.001**	64 (48 – 82)	73 (56 – 88)	** < 0.001**	79 (56 – 88)	** < 0.001**	0.195	0.085	0.793
FVC % of predicted	82 (70 – 94)	90 (76 – 104)	** < 0.001**	91 (75 – 102)	** < 0.001**	79 (64 – 91)	91 (76 – 103)	** < 0.001**	91 (77 – 103)	** < 0.001**	81 (69 – 95)	90 (76 – 99)	** < 0.001**	91 (77 – 105)	** < 0.001**	0.123	0.067	0.557
RV % of predicted	140 (116 – 173)	126 (109 – 154)	** < 0.001**	127 (109 – 155)	** < 0.001**	136 (113 – 159)	127 (112 – 145)	**0.001**	124 (111 – 157)	**0.003**	134 (112 – 161)	129 (110 – 154)	**0.002**	130 (110 – 157)	**0.009**	0.334	0.987	0.320
TLC % of predicted	105 (95 – 116)	107 (97 – 114)	0.462	107 (96 – 115)	0.407	103 (92 – 109)	104 (90 – 116)	0.154	104 (95 – 112)	0.107	101 (93 – 112)	102 (96 – 113)	0.107	103 (97 – 113)	**0.043**	0.430	0.486	0.147
ACT score	13 (10 – 19)	20 (15 – 23)	** < 0.001**	19 (15 – 23)	** < 0.001**	14 (10 – 20)	21 (14 – 25)	** < 0.001**	21 (15 – 24)	** < 0.001**	16 (11 – 19)	19 (15 – 22)	** < 0.001**	20 (15 – 23)	** < 0.001**	**0.033**	0.741	0.087
Exacerbations per year	1 (0 – 2)	0 (0 – 1)	** < 0.001**	0 (0 – 0)	** < 0.001**	1 (0 – 3)	0 (0 – 1)	** < 0.001**	0 (0 – 0)	** < 0.001**	0 (0 – 1)	0 (0 – 1)	0.338	0 (0 – 0)	0.491	**0.003**	0.679	**0.004**
Eosinophils per µl	430 (70 – 740)	50 (20 – 100)	** < 0.001**	30 (1 – 90)	** < 0.001**	400 (63 – 678)	0 (0 – 10)	** < 0.001**	0 (0 – 1)	** < 0.001**	220 (60 – 475)	390 (153 – 738)	0.114	9 (3 – 330)	**0.025**	** < 0.001**	** < 0.001**	0.744
FeNO in ppb	36 (19 – 71)	35 (19 – 58)	**0.011**	34 (23 – 57)	**0.024**	42 (23 – 65)	38 (18 – 66)	0.123	31 (16 – 62)	0.200	36 (21 – 56)	22 (16 – 31)	** < 0.001**	21 (16 – 29)	** < 0.001**	**0.017**	0.936	**0.033**
OCS dose	5 (0 – 10)	0 (0 – 4)	** < 0.001**	0 (0 – 3.75)	** < 0.001**	2.5 (0 – 7.5)	0 (0 – 5)	**0.07**	0 (0 -5)	** < 0.001**	0 (0 -5)	0 (0 – 0)	**0.036**	0 (0 – 0)	0.055	0.353	0.377	0.836

*Abbreviations*: *IQR *Interquartile range, *T1 *Follow-up timepoint 1 (6 months), *T2 *Follow-up timepoint 2 (12 months), *FEV1 *Forced expiratory capacity in 1 s, *FVC *Forced vital capacity, *RV *Residual volume, *TLC *Total lung capacity, *ml *milliliter, *ACT *Asthma control test, *OCS *Oral corticosteroids, *FeNO *Fractional expiratory nitric oxide, *ppb *Parts per billion

^a^Intra-group *p*-values calculated using t-tests

^b^Inter-group *p*-values comparing delta changes between treatment groups calculated using two-way ANOVA with Bonferroni-correction

Both at T1 and T2, all groups showed significant improvements in PF (Fig. 1C and D). Notably, blood eosinophils decreased significantly in patients receiving mepolizumab or benralizumab from BL to T1, but patients receiving dupilumab showed a significant decrease in eosinophils only at T2 (Fig. 1E). FeNo decreased significantly in patients treated with either mepolizumab or dupilumab, but not benralizumab.

At baseline, roughly half of all patients in our cohort received OCS therapy (mepolizumab: 58%, benralizumab: 56%, dupilumab: 43%, p = 0.095). The median OCS dosage decreased significantly from baseline in all groups, despite starting at relatively low average baseline dosages in all groups. In particular, the mean and median dosage of OCS in the dupilumab group was comparatively low, with limited room for further reduction. By consequence, the OCS reductions from BL to T1 were significantly stronger in patients receiving mepolizumab or benralizumab. (Table 2; Fig. 1F).

**Fig. 2:**
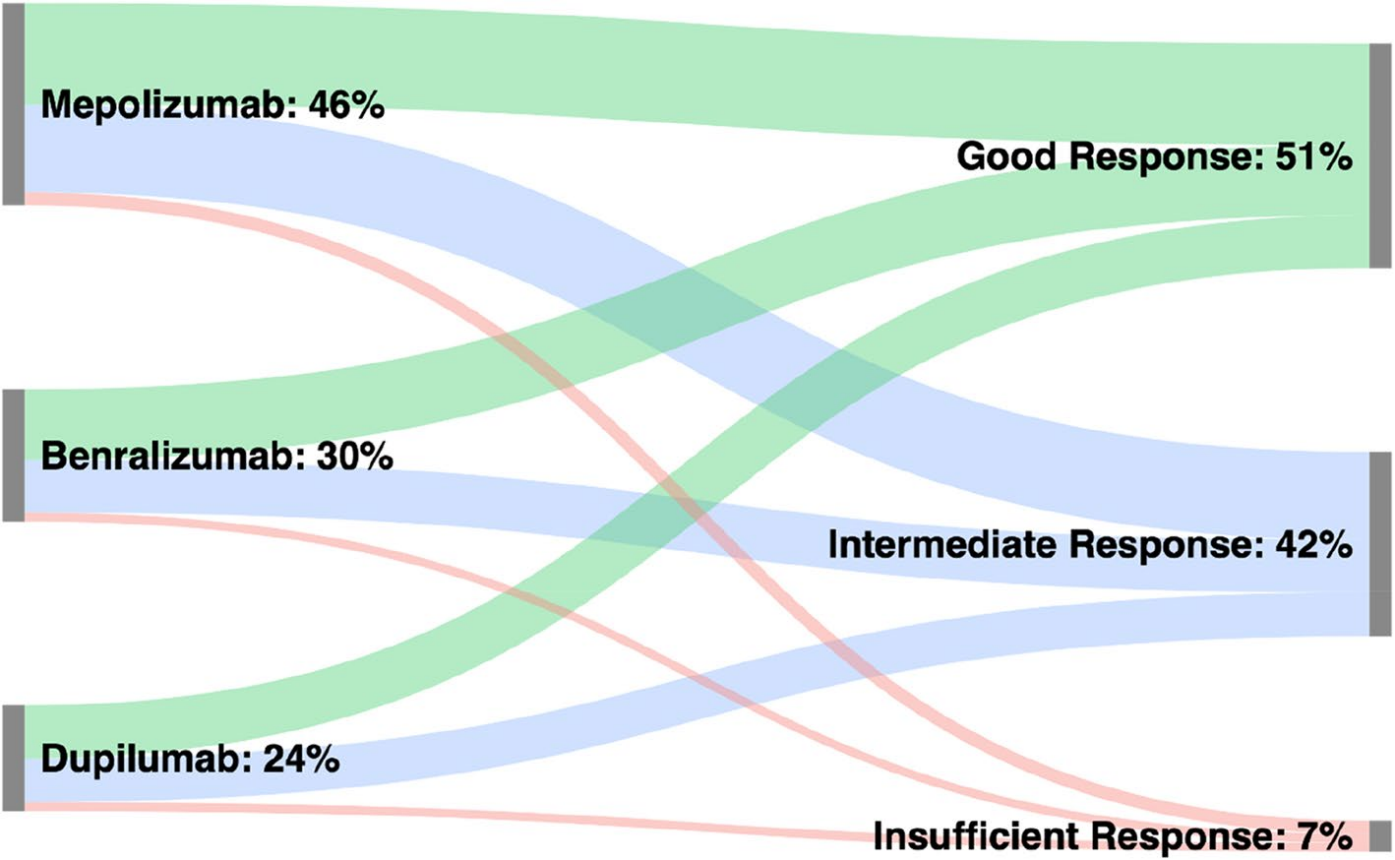
Therapy response assessed by BARS after one year. Notes: Sankey chart shows responder status according to BAR-score after one year of biologic therapy. Percentages are calculated in relation to all patients with complete datasets in the study. BARS – Biologics Asthma Response Score

Response to biologic therapy was calculated for both timepoints using BARS. Therapy response was strong across all groups, with roughly 50% of patients attaining good therapy response, and the majority of the remainder showing at least intermediate therapy response across all groups. This effect was attained by T1 and continued unaltered by T2, without significant differences between the groups. Results are summarized in Table 3 and Fig. 2.

**Table 3:** BARS responder classification

	**Mepolizumab, *n* = 129**	**Benralizumab, *n* = 83**	**Dupilumab, *n* = 68**	**Inter-group comparison**
**BARS Score at V1 (6 months)**				
Good Response, n (%)	65 (50)	37 (45)	33 (49)	0.460
Intermediate Response, n (%)	45 (35)	33 (40)	25 (37)	
Insufficient Response, n (%)	6 (5)	9 (11)	7 (10)	
Data incomplete, n (%)	13 (10)	4 (5)	3 (4)	
**BARS Score at V2 (12 months)**				
Good Response, n (%)	64 (50)	44 (53)	35 (51)	0.928
Intermediate Response, n (%)	57 (44)	33 (40)	27 (40)	
Insufficient Response, n (%)	6 (5)	5 (6)	6 (9)	
Data incomplete, n (%)	2 (1)	1 (1)	–	

Comparative BARS response distribution calculated using Chi^2^ test. BARS—Biologics Asthma Response Score, T1 – follow-up timepoint 1 (6 months), T2 – follow-up timepoint 2 (12 months)

## Discussion

In this multi-centered, retrospective study, we evaluated the clinical efficacy and rate of therapy response in long-term biologic therapy with either mepolizumab, benralizumab or dupilumab in 280 severe asthma patients in a real-life setting. Overall, we observed excellent efficacy and high rates of therapy response, both regarding therapy assessment by BARS and individual parameters like PF in all three treatment groups. These beneficial effects were usually fully established after 6 months and remained consistent after 12 months.

The portfolio of biologics for the treatment of severe asthma with type-2 inflammation has expanded significantly in the past decade, yet the question which biologic is the most effective remains unanswered. Previous work re-analyzing existing licensing-trial data comparing the efficacy of anti-IL5 and anti-IL5Rα biologics led to conflicting results, observing either no differences or and advantage of mepolizumab in improving asthma control and exacerbation reduction. Notably, the latter observation was not confirmed in a previous retrospective analysis of real-life patient data by our group [15, 16, 18]. Either way, the above-mentioned publications are based on analyses of existing phase-3 drug trial data via an indirect comparison between treatment groups. Inevitably, this pre-existing licensing trial data represents a heavily pre-selected sample that cannot be considered entirely representative of the patient cohort faced by physicians in clinical practice [15]. To bridge this gap in the existing data, our study used real-life clinical data, representing a patient sample more closely resembling the average severe asthma patient. In addition, our approach allows for a more direct efficacy comparison. Notably, all three groups showed largely similar baseline characteristics, apart from a higher prevalence of asthma-related comorbidities like CRSwNP and atopic dermatitis in the dupilumab group. At the same time, we observed lower rates of CRSwNP compared to other cohorts, independent of treatment group [28–31]. However, the comparatively increased rate of CRSwNP and atopic dermatitis in dupilumab recipients in our cohort is unsurprising, given the well-established additional benefits of this antibody in the co-treatment of these atopic phenotype diseases. While the ideal way to phenotype asthma is still up for debate and definitions are frequently subject to change, the distinction between a more allergic asthma phenotype, an eosinophilic phenotype and a mix thereof is one of the most commonly used clinical distinctions both inside and outside of clinical guidelines [32]. In this context, it is worth noting that the dupilumab cohort in our study featured more patients with a mix of allergic and eosinophilic asthma phenotypes. This distribution likely is the effect of intentional prescription choices in our clinical practice, as we had previously observed a particular advantage of dupilumab therapy among patients with a mixed asthma phenotype [33]. We observed a comparatively high rate of ex-smokers in our cohort compared to other studies [34, 35]. However, Germany has the fourth highest smoking rate in the European Union, likely explaining the high rate of ex-smokers even among severe asthma patients [36].

Antibody therapy led to significant improvements across nearly all assessed parameters in all three treatment groups. All lung function parameters except for TLC improved significantly in all groups, indicating improved pulmonary performance. Notably, these improvements were comparable in all treatment groups, without significant differences. Furthermore, both asthma control as measured by ACT, annualized exacerbation rate and the need for and average dosage of OCS improved in all treatment groups, highlighting the tangible clinical benefits of biologic therapy in severe asthma patients. However, these reductions were statistically significantly more pronounced among patients receiving either mepolizumab or benralizumab, compared to patients receiving dupilumab. However, this is unlikely to represent a clinically significant difference, as both the annualized exacerbation rate and mean OCS dose were lower in the dupilumab cohort at baseline, thus allowing for less overall reduction upon therapy initiation. Likewise, patients receiving dupilumab had a slightly higher average ACT score at baseline. While all patients in our cohort suffered from severe asthma insufficiently controlled by conventional therapy, a prerequisite to biologic therapy initiation according to German guidelines (Supplementary Table 1) [24], the less severe baseline impairments indicate that patients in the dupilumab group had better asthma control at baseline, and thus perhaps less room for improvement. Notably, prescription guidelines for biologics in severe asthma are heterogenous among European countries. In the future, unified international prescription criteria would be desirable to facilitate international comparisons [37].

In terms of laboratory parameters, we observed some divergence: while the dupilumab cohort showed a less pronounced decrease in peripheral blood eosinophils at T1 compared to the other treatment groups, this distinction was lost by T2. This confirms the well-described observation that dupilumab may induce a transient increase in eosinophils prior to a reduction to baseline or below, but without affecting clinical efficacy of the drug [38]. Additionally, we observed statistically significant reductions in FeNo, an important marker for airway inflammation in patients receiving either mepolizumab or dupilumab, but not in those receiving benralizumab. While FeNo has been described as a sensitive and cost-effective biomarker with the potential ability to predict exacerbations, its predictive value in the setting of patients already receiving maximized asthma therapy is less clear [39]. Given the equally strong effects of benralizumab on all other clinical parameters in our study, a clinically relevant effect of this less pronounced reduction in our setting is unlikely. Additionally, while FeNo levels decreased statistically significantly among mepolizumab recipients, the biological relevance of this finding is unclear given the minimal absolute FeNo decreases in this group.

While many studies have analyzed potential benefits of asthma therapy using a plethora of parameters, comparisons have so far been hampered by the absence of universally accepted scoring systems for biologic therapy response. Recently, a German consensus group published the BAR-Score, a scoring system for biologic therapy response in severe asthma allowing for easier and more objective treatment comparisons [22]. Calculated through assessment of ACT, annualized exacerbation rate and reduction of steroid dose, BARS allows for categorization of biologic recipients into “good response”, “intermediate responders” and “insufficient response” [22]. Using BARS, we found that around half of all patients attained a good response, with more than another third showing intermediate response, irrespective of group. We did not observe lacking responses being driven by any individual factor (ACT, exacerbations, or OCS) for any specific biologic. Simultaneously, this underlines both the overall good efficacy of biologics and the utility of the novel BAR-score in therapy assessment. Given the increased importance of “super-responders” and the complete asthma remission in biologic therapy in severe asthma, it is important to emphasize that BARS response, “super-response” and “asthma remission” fit similar, but not identical criteria. While there are multiple concurrent definitions of superresponse currently proposed, it is safe to assume that independent of definition all supperresponders and patients in asthma remission would be BARS responders, but not all BARS responders might fit the “superresponder” or “remission” label [40, 41].

Given the chronic nature of asthma and seasonal variations of symptoms and exacerbations, treatment efficacy should be assessed with a long-term perspective in mind. This aspect complicates the interpretation of the various licensing trials, not least due to the variable time-frames used, ranging from 32 weeks in the MENSA trial (mepolizumab) to 52 weeks and 56 weeks in the LIBERTY ASTHMA QUEST (Dupilumab) and CALIMA (benralizumab) trials [42–44]. To better account for seasonality of exacerbations, particularly in conjunction with infections and seasonal allergens, we chose to assess a full year of asthma therapy [45]. In line with the current GINA and NVL guidelines, as well as previous findings by our group and others, we observed that most patients had already achieved their maximum treatment response around 6 months of therapy, while retaining their level of response at the later timepoint [21, 27, 46]. At the same time, this underlines the utility of a therapy switch attempt, potentially supported through BARS therapy assessment, in those patients with unsatisfactory therapy response after 4–6 months [23, 33].

### Limitations

The retrospective design of our study limits its generalizability and may have introduced some confounding factors, not least of which may be the disbalance in group sizes due to the longer market availability of some antibodies. In particular, this may have favored the mepolizumab group with regards to statistical significances. Moreover, market availability at the time influenced antibody treatment choices and we cannot exclude that some patients who were treated with an earlier marketed antibody (i.e. mepolizumab) would have been phenotypically eligible for a different, newer antibody (i.e. dupilumab) and would have been treated with that antibody, had it been available at the time. Furthermore, as we intended to investigate the long-term treatment efficacy of mepolizumab, benralizumab and dupilumab, only patients who had completed a full year of antibody therapy were eligible for this study. By consequence, our analysis may overestimate the efficacy of either antibody both in terms of individual parameters and BARS, as patients who benefited are more likely to have completed a full year of therapy. Given that we observed around 7% of “insufficient responders” and a sizable group of only “intermediate responders” as evaluated by BARS despite this selection bias, it is however unlikely that our study population was affected unduly. Furthermore, this possible bias is partially offset in so far as it would affect all three treatment groups and thus should not impact comparisons between the antibodies. Lastly, we were unable to include the latest addition to the biologics portfolio for asthma therapy, the anti-TSLP-antibody Tezepelumab, as it has only been approved by the European Medicines Agency in the fall of 2022, and thus 12 month data was not yet available at the time of study conclusion.

## Conclusion

The data we present supports earlier reports by our group and others, emphasizing the great and largely similar clinical efficacy of the available biologics for asthma therapy [15, 16, 21]. All antibodies led to solid and persistent improvements, both in terms of individual parameters like PF and in terms of BAR-Score response. Nonetheless, a considerable percentage of patients did not respond optimally to therapy, emphasizing the need for further research into ideal switching strategies in non-responders, as well as potential predictive factors to help guide the antibody selection process in asthma therapy.

### Supplementary Information

**Supplementary material 1.**

## Data Availability

The datasets used and/or analyzed during the current study are available from the corresponding author on reasonable request within 5 years of publication.

## Abbreviations

ACT: Asthma control test
ATS: American thoracic society
BARS: Biologic Asthma Response Score
BL: Baseline
COPD: Chronic obstructive pulmonary disease
ERS: European respiratory society
FeNO: Fractional exhaled nitric oxide
FEV1: Forced expiratory volume in one second
FVC: Functional vital capacity
ICS: Inhaled corticosteroids
IL: Interleukin
IL5Rα: Interleukin 5 receptor alpha
ILC2: Innate lymphoid cell type 2
IQR: Interquartile range
OCS: Oral corticosteroids
PF: Pulmonary function
RV: Residual volume
SD: Standard deviation
T1: Timepoint 1
T2: Timepoint 2
TLC: Total lung capacity
