# Supplementary material for: Long-term multicenter comparison shows equivalent efficacy of monoclonal antibodies in severe asthma therapy
Source: BMC Pulm Med. 2024 Mar 21;24:149. doi: 10.1186/s12890-024-02964-4 (PMC10956233; doi:10.1186/s12890-024-02964-4)
Supplement: Supplementary file 1 — Supplementary material 1. [file 12890_2024_2964_MOESM1_ESM.pdf]

General prescription criteria:		
Severe asthma treated with medium-high dose ICS, LABA and OCS or other 2nd/3rd controller		
Insufficient asthma control (based on exacerbations, permanent OCS-dependency, AQL)		
**Specific prescription criteria**		
**Mepolizumab**	**Benralizumab**	**Dupilumab**
Severe eosinophilic asthma	Severe eosinophilic asthma	Severe asthma
>/= 300 eosinophils /µl in last 12 months (outside of exacerbation)	>/= 300 eosinophils /µl in last 12 months (outside of exacerbation)	FeNO >/= 25 ppb in last 12 months **and/or**
>/= 150 eosinophils / µl in last 12 months (outside of exacerbation) if ongoing OCS therapy	>/= 150 eosinophils / µl in last 12 months (outside of exacerbation) if ongoing OCS therapy	>/= 150 eosinophils / µl in last 12 months
Additional prescription criteria for co-morbidities:<br>- EGPA<br>- HES<br>- CRSwNP	Additional prescription criteria for co-morbidities:<br>- none	Additional prescription criteria for co-morbidities:<br>- Neurodermitis<br>- CRSwNP<br>- EoE<br>- PN

ICS – inhaled corticosteroids, LABA – long-acting beta agonists, OCS – oral corticosteroids, AQL – asthma-related quality of life, CRSwNP - Chronic Rhinosinusitis with Polyposis, EGPA: Eosinophilic Granulomatosis with Polyangiitis, EoE – Eosinophilic Esophagitis, HES – Hypereosinophilic Syndrome, PN - Prurigo nodularis.
